# Dental Effects of Discordant Thumb‐Sucking in Monozygotic Twins: A Case Report

**DOI:** 10.1155/crid/8643566

**Published:** 2026-02-27

**Authors:** Kevin Brunstein, Eric Hu, Jillian Shute

**Affiliations:** ^1^ Department of Pediatric Dentistry, U.S. Army Fort Hood 2-Year Advanced Education in General Dentistry, Army Post Graduate Dental School, Fort Hood, Texas, USA; ^2^ Department of Prosthodontics, U.S. Army Fort Hood 2-Year Advanced Education in General Dentistry, Army Post Graduate Dental School, Fort Hood, Texas, USA; ^3^ Department of Orthodontics, U.S. Army Fort Hood 2-Year Advanced Education in General Dentistry, Army Post Graduate Dental School, Fort Hood, Texas, USA

**Keywords:** case report, monozygotic twins, nonnutritive sucking habit, primary dentition, thumb-sucking

## Abstract

This case study examines the dental effects of a persistent thumb‐sucking habit by comparing monozygotic twins—one with the habit and one without. With genetic and environmental variables largely controlled, this model isolates the impact of nonnutritive sucking on occlusal development. Clinical and diagnostic assessments revealed that the twin with the habit exhibited an anterior open bite, increased overjet, narrower maxillary arch width, deeper palatal vault, and altered incisor inclinations. A unilateral right‐sided functional shift crossbite was also observed. Molar relationships were similar in both twins, whereas canine relationships differed. These findings align with existing literature on the association between digit habits and malocclusion. This genetically controlled comparison strengthens the evidence that persistent thumb‐sucking can influence the developing dentition and contributes to a deeper understanding of behavioral influences on occlusal and arch morphology in children in the primary dentition.

## 1. Introduction

Infants exhibit natural and instinctive sucking behavior that serves physiological and psychological purposes [1, 2, 3]. Although nutritive sucking, such as breastfeeding or bottle feeding, fulfills the infant′s nutritional needs, nonnutritive sucking habits (NNSH), including thumb, finger, and pacifier use, are commonly employed for self‐soothing, emotional regulation, and stress relief [1, 3]. NNSH are considered developmentally normal in infancy and early childhood [3, 4, 5]. Studies suggest that approximately 40% of children will continue to have a NNSH at age 3 [5]. The average age at which digit sucking ceases spontaneously is between 3 and 4 years of age [4, 6]. Most of the remaining children discontinue the habit by the time they enter the early mixed dentition phase [4, 6].

Although early digit sucking typically has minimal long‐term effects, persistence of the habit beyond age 4 increases the risk of developing long‐term effects on the dentition [4, 7]. Studies have documented an association between prolonged digit habits and alterations in dental and skeletal development including anterior open bite, increased overjet, posterior crossbite, and transverse maxillary constriction [2, 4, 8, 9]. Anterior open bite results from the placement of the thumb or finger between the maxillary and mandibular incisors, which interferes with their vertical eruption and allows for supraeruption of the molars [4, 5]. In addition, the pressure exerted by the digit may lead to labial proclination of the maxillary incisors and lingual retroclination of the mandibular incisors [9].

Transverse discrepancies such as posterior crossbite have been linked to digit habits through the displacement of the tongue leading to unopposed inward pressure of the buccal musculature [9, 10]. This muscular imbalance may result in the collapse of the maxillary arch [8–10]. Several studies have demonstrated the relationship between prolonged sucking habits and maxillary constriction or crossbite [2, 8, 11].

The severity of the dental changes associated with NNSH is influenced by multiple factors, including the duration of the habit (in months/years), daily frequency (hours per day), intensity of the applied force (force magnitude), and direction of pressure (force vector) [4, 5, 12]. Of these, duration appears to have the most critical impact on the dentition [5, 12]. Children who apply intermittent but intense force may not exhibit the same degree of change as those who exert mild pressure over prolonged periods [12]. Persistent habits that extend beyond the eruption of permanent incisors can lead to dental discrepancies and malocclusions that are less likely to self‐correct after the habit is eliminated [4, 5]. These discrepancies will often require orthodontic intervention [4, 5].

The association between NNSH and malocclusion is well documented; however, individual variability in dental response, likely influenced by genetic factors, can complicate the interpretation of causality [7, 13]. Although traditional observational studies consistently demonstrate correlations between oral habits and dental changes, differences in growth patterns and skeletal morphology may act as confounding variables. In this context, monozygotic twin comparisons offer a unique model to control for hereditary influences and isolate the specific contribution of the NNSH. Monozygotic twins are genetically identical siblings who develop from a single fertilized egg that splits into two embryos. This unique genetic similarity allows for isolated examination of environmental influences, such as oral habits, on dental development. Despite the potential value of this approach, the literature contains few such case studies [14].

The objective of this report is to provide a comparative analysis of the primary dentition in a pair of monozygotic twins, one with a persistent thumb‐sucking habit and the other without. By examining differences in occlusion, arch width, palatal depth, and incisor inclination, this case study helps contribute to a better understanding of the specific effects of prolonged thumb‐sucking on the primary dentition.

## 2. Case Report

Monozygotic twins, referred to as Twin H (with a thumb‐sucking habit) and Twin N (no habit), both aged 5 years and 11 months, presented for evaluation with the Pediatric Dentistry Department of the U.S. Army Fort Hood Advanced Education in General Dentistry Program. The twins were born prematurely at 27 weeks gestation and required an extended stay in the neonatal intensive care unit (NICU). At the time of examination, both were developing within normal limits and following appropriate growth curves.

Medical histories revealed that both twins had been diagnosed with mild persistent asthma and seasonal allergic rhinitis. They were being managed under the care of their pediatrician and followed identical medical regimens, including daily use of montelukast (administered twice daily) and fluticasone propionate (two puffs twice daily via spacer). Each child also used an albuterol rescue inhaler as needed for bronchospasm and took cetirizine on an as‐needed basis for seasonal allergy symptoms

The parents of the twins reported that Twin H had a longstanding and persistent thumb‐sucking habit, which had been present since infancy. The habit was active for the majority of the day, with the exception of mealtimes, and was visibly evident during the dental visit. Notably, her right thumb appeared cleaner, wrinkled, and moist compared with her other fingers. Her typical thumb‐sucking posture involved placement of the right thumb such that the lips rested midway between the interphalangeal and metacarpophalangeal joints, with the palmar surface oriented toward the palate. Previous parental attempts to eliminate the habit, including behavioral incentives, positive reinforcement, thumb wrapping, and application of bitter‐tasting nail polish, had been unsuccessful. No other nonnutritive sucking or oral habits were reported. In contrast, Twin N had no history of thumb or finger sucking, and parents denied awareness of any other nonnutritive sucking behaviors or oral habits.

**Figure 1:**
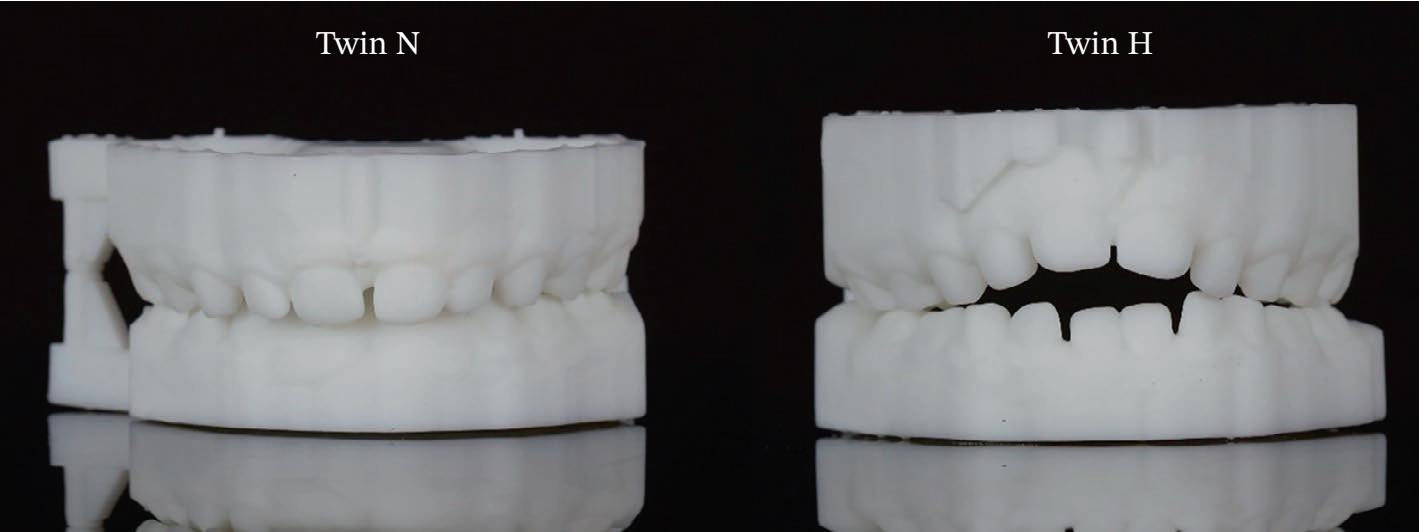
Frontal views of Twin N and Twin H in maximum intercuspation.

**Figure 2:**
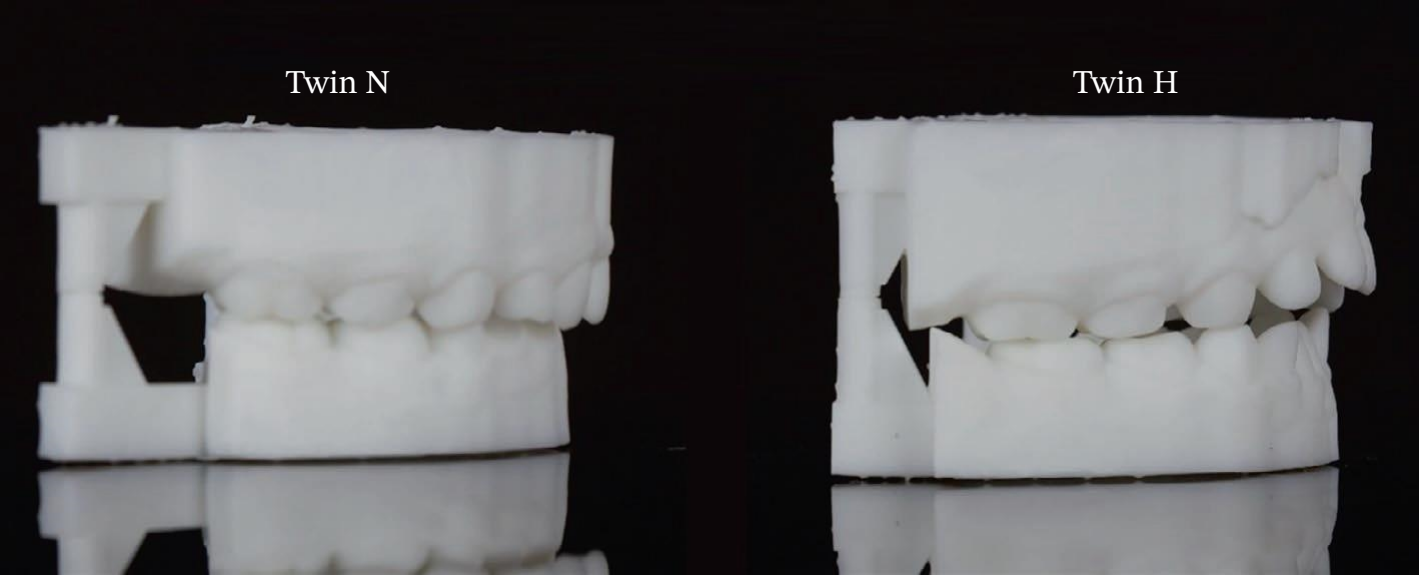
Right buccal views of Twin N and Twin H in maximum intercuspation.

**Figure 3:**
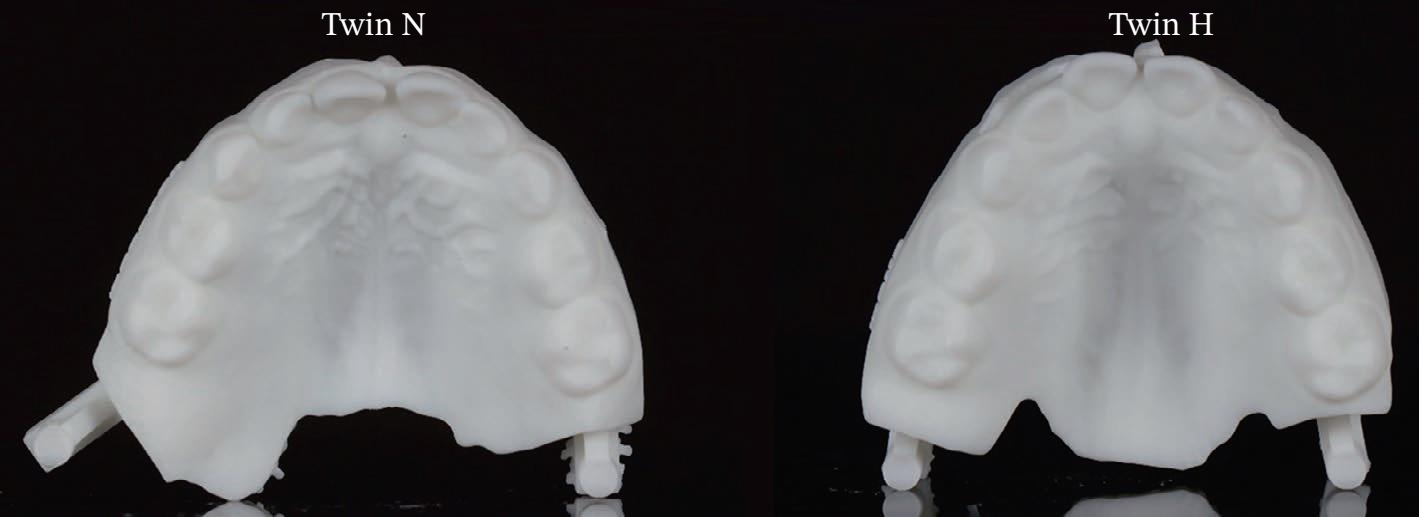
Maxillary occlusal views of Twin N and Twin H.

Both twins underwent comprehensive dental evaluations, including extraoral and intraoral examinations, radiographic assessments, and digital intraoral scanning using the CEREC Primescan (Dentsply Sirona Inc., Charlotte, North Carolina). Diagnostic casts were fabricated via 3D printing using a biocompatible resin (White Resin, Formlabs Inc., Somerville, Massachusetts) on an SLA printer (Form 3B, Formlabs Inc., Somerville, Massachusetts). The study casts were examined side‐by‐side, and quantitative measurements of dental arch morphology and occlusion were obtained using a calibrated digital caliper (ProDentUSA, East Brunswick, New Jersey). Results are presented in Table 1 with corresponding clinical photographs shown in Figures 1, 2, and 3.

**Table 1:** Comparison of dental findings.

	Twin N (no habit)	Twin H (habit)
Overbite	6.04 mm	−2.75 mm
Overjet	2.84 mm	6.01 mm
Maxillary intercanine width	29.80 mm	28.08 mm
Maxillary intermolar width (ML cusp)	36.23 mm	33.63 mm
Depth of palate (measured from a perpendicular line dropped from molars ML cusp)	12.00 mm	16.73 mm
Mandibular intercanine width	24.70 mm	24.29 mm
Mandibular intermolar width (central fossa)	40.13 mm	38.28 mm
Right molar relationship	Mesial step	Mesial step
Right canine relationship	Angle′s Class I	Angle′s Class II
Left molar relationship	Mesial step	Mesial step
Left canine relationship	Angle′s Class I	Angle′s Class II
Posterior crossbite	No	Right side–functional shift
Other findings	Mandibular incisors retroclined	Maxillary incisors proclined, mandibular incisors retroclined

## 3. Discussion

The comparative evaluation of monozygotic twins in this case provides an opportunity to isolate the effects of a persistent thumb‐sucking habit on dental development. Despite identical genetic makeup and shared medical and environmental factors, Twin H, who maintained a prolonged thumb‐sucking habit, exhibited several clinically significant deviations from her sibling, Twin N, who had no history of nonnutritive sucking behaviors. These findings highlight the substantial impact of prolonged oral habits on the primary dentition.

One of the most striking findings was an overbite difference of nearly 9 mm between the twins. Twin H demonstrated an anterior open bite (overbite of −2.75 mm) compared with Twin N′s positive overbite (6.04 mm). Twin H also demonstrated about 3 mm greater overjet. These changes, visible in frontal intraoral views (Figure 1) and right buccal view (Figure 2), are consistent with anterior open bite and incisor flaring. The findings likely result from mechanical interference with vertical eruption and prolonged anterior pressure from thumb placement.

Transverse differences further underscore the environmental impact of the habit. Twin H′s maxillary intermolar width was 2.6 mm narrower and her palatal vault was 4.7 mm deeper than Twin N′s. These differences are illustrated in the maxillary occlusal views (Figure 3). Twin H also presented with a right‐sided functional shift crossbite, likely reflecting an emerging transverse discrepancy secondary to maxillary constriction. These measurements suggest vertical remodeling and maxillary arch constriction. These changes suggest that chronic thumb‐sucking may have displaced the tongue inferiorly, eliminating its expansive influence on the maxillary arch and contributing to vertical remodeling of the palatal vault. This pattern supports the equilibrium theory, which posits that dental arch form is governed by the balance of forces from the lips, cheeks, and tongue [9].

Although both twins exhibited bilateral mesial step molar relationships, Twin H exhibited bilateral Angle′s Class II canine relationships, in contrast to Twin N′s bilateral Angle′s Class I. This divergence may reflect developing anteroposterior discrepancies secondary to prolonged thumb‐sucking, which likely influenced arch development and altered the position of the canines.

This case strengthens the evidence that thumb‐sucking can result in measurable and clinically significant effects on the developing dentition, independent of genetic variability. The monozygotic twin model offers a valuable framework for isolating environmental influences and may support earlier recognition of habit‐related changes in clinical practice. Limitations to this study include observing only a single monozygotic twin pair, possible residual environmental confounders, parent‐reported habit exposure without objective quantification, no cephalometric imaging or reliability testing, and no longitudinal follow‐up, which limits causal inference and generalizability. Future research involving larger twin cohorts, longitudinal follow‐up into the mixed and permanent dentition, and assessment of habit cessation interventions would provide further clarity on the long‐term effects and management of NNSH.

## 4. Conclusion

In this monozygotic twin comparison, the child with a persistent thumb‐sucking habit exhibited anterior open bite, increased overjet, maxillary transverse constriction, a deeper palatal vault, and a unilateral functional shift crossbite, whereas the co‐twin without the habit did not. Clinically, these findings underscore the need for early recognition of anterior, transverse, and vertical changes associated with NNSH, proactive parent counseling on habit cessation, and timely interceptive management when indicated.

## Author Contributions

**Kevin Brunstein:** conceptualization, methodology, investigation, data curation, analysis, writing – original draft, and project administration. **Eric Hu:** formal analysis, visualization, and writing – review and editing. **Jillian Shute:** formal analysis, visualization, and writing – review and editing.

## Funding

The authors received no financial support for the research, authorship, and/or publication of this article.

## Disclosure

This manuscript was reviewed and cleared for public release by the Fort Hood Public Affairs Office (PAO) on May 2, 2025. No protected health information is disclosed. The identification of specific products, scientific instrumentation, or organization is considered an integral part of the scientific endeavor and does not constitute endorsement or implied endorsement on the part of the authors, Department of Defense, or any component agency. The views expressed in this material are those of the authors and do not reflect the official policy or position of the Department of the Army, Uniformed Services University, Department of Defense, or the US Government.

## Ethics Statement

This case report did not require institutional review board approval as it describes routine clinical care without experimental interventions or identifiable patient information.

## Consent

Written informed consent for publication of this case report and accompanying images was obtained from the patients′ legal guardian. Images have been deidentified and cropped.

## Conflicts of Interest

The authors declare no conflicts of interest.

## Data Availability

All data supporting the findings of this case report are included within the article. Additional deidentified data are available from the corresponding author on reasonable request.
